# Long-Term Effects of Red- and Blue-Light Emitting Diodes on Leaf Anatomy and Photosynthetic Efficiency of Three Ornamental Pot Plants

**DOI:** 10.3389/fpls.2017.00917

**Published:** 2017-05-30

**Authors:** Liang Zheng, Marie-Christine Van Labeke

**Affiliations:** Department of Plant Production, Ghent UniversityGent, Belgium

**Keywords:** leaf anatomy, leaf hydraulic conductance, chlorophyll fluorescence, stomatal conductance, *Ficus benjamina*, *Sinningia speciosa*, *Cordyline australis*

## Abstract

Light quality critically affects plant development and growth. Development of light-emitting diodes (LEDs) enables the use of narrow band red and/or blue wavelengths as supplementary lighting in ornamental production. Yet, long periods under these wavelengths will affect leaf morphology and physiology. Leaf anatomy, stomatal traits, and stomatal conductance, leaf hydraulic conductance (K_leaf_), and photosynthetic efficiency were investigated in three ornamental pot plants, namely *Cordyline australis* (monocot), *Ficus benjamina* (dicot, evergreen leaves), and *Sinningia speciosa* (dicot, deciduous leaves) after 8 weeks under LED light. Four light treatments were applied at 100 μmol m^−2^ s^−1^ and a photoperiod of 16 h using 100% red (R), 100% blue (B), 75% red with 25% blue (RB), and full spectrum white light (W), respectively. B and RB resulted in a greater maximum quantum yield (F_v_/F_m_) and quantum efficiency (Φ_PSII_) in all species compared to R and W and this correlated with a lower biomass under R. B increased the stomatal conductance compared with R. This increase was linked to an increasing stomatal index and/or stomatal density but the stomatal aperture area was unaffected by the applied light quality. Leaf hydraulic conductance (K_leaf_) was not significantly affected by the applied light qualities. Blue light increased the leaf thickness of *F. benjamina*, and a relative higher increase in palisade parenchyma was observed. Also in *S. speciosa*, increase in palisade parenchyma was found under B and RB, though total leaf thickness was not affected. Palisade parenchyma tissue thickness was correlated to the leaf photosynthetic quantum efficiency (Φ_PSII_). In conclusion, the role of blue light addition in the spectrum is essential for the normal anatomical leaf development which also impacts the photosynthetic efficiency in the three studied species.

## Introduction

Light strongly influences plant growth and development. Light, as an energy source, affects photosynthesis and its related parameters. Light quality is one of the main factors of light signaling and affects numerous processes from seed germination, leaf formation to flower development (Hogewoning et al., 2010; Wang et al., 2010; Johkan et al., 2012; Demotes-Mainard et al., 2016). Artificial lighting has been used to extend the photoperiod and to increase the light intensity in horticultural production. Development of light-emitting diodes (LEDs) enables the application of narrow spectrum band red or blue wavelengths in the cultivation of horticultural crops at the exact absorption peaks of chlorophyll (Dutta Gupta and Jatothu, 2013) which in short-term results in the highest photosynthetic efficiencies per leaf unit area (McCree, 1971). Yet, long periods under monochromatic or dichromatic wavelengths with low natural light fluencies might lead to many morphological and physiological changes in response to the ambient light environment thus affecting plant development (Terashima and Saeki, 1983; Brodersen and Vogelmann, 2010; Demotes-Mainard et al., 2016; Huché-Thélier et al., 2016).

Various traits affecting photosynthesis are influenced by light quality including both red and blue light responses. Leaf anatomy may directly influence light capture by its leaf thickness as well as by the differentiation of palisade and spongy mesophyll. Schuerger et al. (1997) reported that leaf thickness increased when red light was supplemented with blue light. Light absorption will also be dependent on chlorophyll concentration. Wang et al. (2009) reported that blue light enhanced the expression of different enzymes such as MgCH, GluTR, and FeCH which regulate the synthesis of chlorophyll. Red light is less conducive for the chlorophyll biosynthesis, because of its reduction of the tetrapyrrole precursor 5-aminolevulinic acid (Sood et al., 2005; Fan et al., 2013). Stomatal density and conductance are other traits that will influence the CO_2_ uptake and thus photosynthesis. Effects of blue light on stomatal opening are well documented (Tallman and Zeiger, 1988; Talbott, 2002). Monochromatic red light has been reported to reduce the photosynthetic efficiency and it leads to photo-damage after a long-term exposure (Trouwborst et al., 2016). In contrast, blue light which is sensed by cryptochrome and phototropin optimizes photosynthesis by improving the efficiency of light capture, reducing photo-damage, and regulating gas exchange between leaves and atmosphere (Takemiya et al., 2005).

Light quality not only affects the gas exchange but also the water transportation within leaves (Sharkey and Raschke, 1981; Lee et al., 2007; Savvides et al., 2012). Leaf hydraulic conductance (K_leaf_) affects different aspects of plant functioning such as respiration, evaporation, and photosynthetic carbon fixation (Prado and Maurel, 2013). Leaf hydraulic conductance reflects the water flow through the leaf veins, across the mesophyll tissue and to the stomatal aperture. The extra-veinal phase of water flow is influenced by the leaf mesophyll spongy/palisade anatomy and thickness and the stomatal aperture characteristics (Nardini et al., 2003; Sack et al., 2004; Sack and Holbrook, 2006). Despite the great importance of leaf hydraulic conductance in plant water relations, knowledge of the relationships between hydraulic conductance and light quality is limited. Savvides et al. (2012) reported that blue in the light spectrum drives both K_leaf_ and g_s_ toward higher values in cucumber. In bur oak, hydraulic conductivity was enhanced in response to blue and green light (Voicu et al., 2008).

In ornamental horticulture, the commercial value depends on the visual quality, which mainly results from architectural traits such as stem elongation, compactness, branching, and flowering. The management of light quality opens the way to improved control of the ornamental value. Control of the light quality by LED lights could also focus on a specific production phase namely the ornamental young plants where LED could be the sole-source light in multilayer production units. However, this phase under monochromatic or dichromatic narrow band LED lights might not only influence the architectural traits but also anatomical traits of leaves developing under this light treatment.

The objective of this study was to evaluate how narrow-band R, B, and RB would modulate leaf morphology, mesophyll anatomy and stomatal formation, which could in consequence influence the light absorption and hydraulic conductance of leaves. To assess the impact on photosynthetic performance chlorophyll fluorescence parameters were quantified as well as the biomass. For this study we selected three commonly produced ornamentals with different leaf traits namely *Cordyline australis* (monocot), *Ficus benjamina* (dicot, evergreen leaves), and *Sinningia speciosa* (dicot, deciduous leaves).

## Materials and methods

### Plant material and growth conditions

The experiment was conducted in a growth chamber at Ghent University, Belgium. Three ornamental species were selected: *C*. *australis* “Red Star” (monocot), *F. benjamina* “Exotica” (dicot, evergreen leaves), and *S. speciosa* “Sonata Red” (dicot, deciduous leaves). Young plants were obtained from a commercial plant producer and transplanted into 0.3 L pots filled with peat-based potting soil (Van Israel nv, Belgium). The plants were acclimated for 1 week in broad spectrum light (100 μmol m^−2^ s^−1^) provided by SON-T high pressure sodium lamps (Philips Inc., Eindhoven, the Netherlands). Then for each species, 12 replicates per treatment were randomly allocated to four spectral light treatments. Air temperature of the growth chamber was set at 22 ± 2°C and plants received a photoperiod of 16 h. Irrigation and fertilization with a water-soluble fertilizer (N:P:K = 4:1:2, EC 1.5 ds m^−1^, pH = 6.5) was applied once every 2 days.

### Light treatment

**Figure 1:**
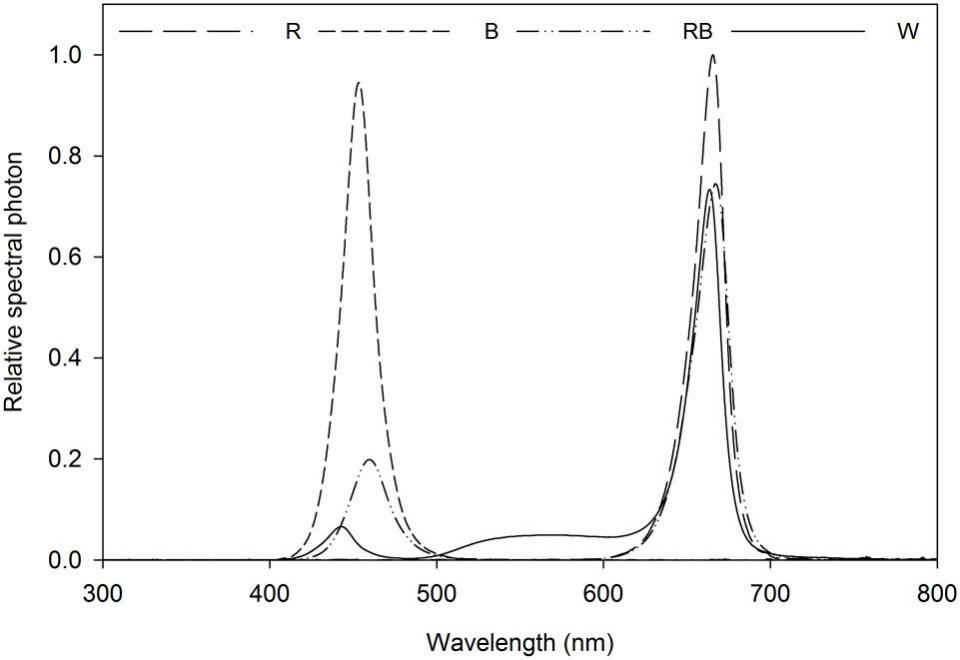
Relative spectral distribution of the light treatments: Red (R), Blue (B), Red/Blue (3:1) (RB) and white (W). Spectrum was measured at the plant canopy level with a JAZ spectrometer (Ocean Optics, FL, US).

Light intensity at the canopy level was set at 100 μmol m^−2^ s^−1^ by adjusting the distance of the light source and a photoperiod of 16 h per day was given. Light treatment sections were separated with curtains, four treatments were applied using different light qualities equipped with LED lighting, which were B (100% blue, peak at 460 nm), R (100% red, 660 nm), and W [white, 7% blue (400–500 nm), 16% green (500–600 nm), 75% red (600–700 nm), and 2% far red (700–800 nm)] (Philips Inc., Eindhoven, The Netherlands) as well as RB (75% R and 25% B, peak at 460 and 660 nm) by a CID-800 programmable LED lighting system (CID Bio-Science, USA), respectively. Light distribution was recorded using JAZ-ULM-200 spectrometer (Ocean Optics, FL, USA) and converted with Spectrasuite software (Ocean Optics) to μmol m^−2^ s^−1^ (Figure 1) and uniformity was verified by measuring the light intensity at five points of each light treatment at the canopy level (Table 1).

**Table 1:** Light treatment with different light spectrum, average PPF per treatment, phytochrome photostationary state (P_fr_/P_total_).

**Parameter**	**R**	**B**	**RB**	**W**
PPFD (400-700 nm) (μmol m^−2^ s^−1^)^a^	97.4 ± 4.2	100.1 ± 1.2	100.3 ± 3.6	97.6 ± 4.7
P_fr_/P_total_^b^	0.884	0.489	0.874	0.879
%B	0	100%	25%	7%

a *Mean ± standard deviation, n = 5*.

b *Phytochrome photostationary state calculated according to Sager et al. (1988)*.

The plants were grown for 8 weeks and then the second or third leaf counting from the apex (fully expanded leaves that developed entirely under the given light quality) were selected for the measurements. All measurements were performed in four replications per treatment and per plant species.

### Leaf anatomy

Leaf segments of 2 × 2 cm of the central leaf blade next to main vein were excised and fixed for at least 24 h in a formaldehyde-based fixative (FAA). Then, leaf segments were dehydrated using a series graded concentration ethanol, embedded in paraffin, and sectioned at thickness of 12 μm with a microtome (R. Jung AG, Heidelberg, Germany). The sections were deparaffinized with xylene and rehydrated with graded ethanol, stained with safranin for 30 min and fast green for 30 s. Stained sections were sealed with Canadian balsam and examined with a bright field microscopy (IX81, Olympus, Japan) at magnification 400x. Images of the cross sections were taken and measured for widths of whole-leaf, palisade mesophyll, spongy mesophyll and abaxial and adaxial epidermal tissues with ImageJ (ImageJ 1.48v, NIH, USA).

### Leaf hydraulic conductivity

The hydraulic conductance of whole leaves (K_leaf_) was performed using a high pressure flowmeter method (HPFM) with slight modifications (Sack et al., 2002). The second or lower fully expanded leaf (depending on the species) was cut next to the petiole stem insertion and immediately placed in a water bath. The petiole was cut under water with a sharp blade to 1 cm length, then wrapped with parafilm (to ensure good seal between petiole and tubing) and inserted into the silicon tube which was connected to the HPFM hydraulic measurement system as described by Tyree (Tyree et al., 2005). Briefly high pressure water was pressed into the leaf vein, leaves were perfused at 0.3 MPa with distilled water for around 60 min until steady-state conditions (±5%), the flow rate was recorded and calculated to leaf hydraulic conductance (mmol m^−2^ s^−1^ MPa^−1^). Leaf area was measured afterward with a leaf area meter (Li-Cor 3000, LiCor, USA) to normalize hydraulic measurements by leaf area.

### Stomatal characteristics and stomatal conductance

Stomatal traits were analyzed using a nail polish print method on the leaf abaxial side as describe by Mott et al. (1991). The nail polish layer was removed with a transparent tape and pasted on a glass slide, the slides were observed with a bright field microscopy (IX81, Olympus, Tokyo, Japan) and stomatal density was calculated based on stomatal counts of 12 microscopic fields (25x, microscopic field 0.16 mm^2^) per leaf, ensuring a 95% confidence level of the results, as the number of stomata per mm^2^. The stomatal index was calculated as number of stomata/(number of epidermal cells + number of stomata) × 100 (Kubinova, 1994). The stomatal aperture, width and length was defined as (Chen et al., 2012) and stomatal aperture area was calculated by assuming an oval pore shape. The total stomatal aperture area per unit leaf area (cm^2^ m^−2^) was calculated as stomatal average density × stomatal aperture area.

Stomatal conductance (g_s_) was measured using a leaf porometer (AP4 porometer, Delta-T Devices, Cambridge, UK). The second/third fully developed leaf (different according to the plant species) was chosen for measurements. Four positions on each leaf were measured and the average result was used as the stomatal conductance of this leaf. *C. australis* is characterized by narrow leaves, which did not allow a correct measurement of g_s_ by porometry, therefore g_s_ of was estimated based on stomatal characteristics as described by Franks and Farquhar (2001):

$$g_{s}=\frac{SDDa^{\prime }}{V\left(l+\frac{\pi }{4}\sqrt{\frac{a^{\prime }}{\pi }}\right)}$$

Where SD = stomatal density (N m^−2^), D = diffusivity of water in air (22°C, 24.5 × 10^−6^ m^2^ s^−1^), a' = stomatal aperture area (m^−2^), V = molar volume of air (m^3^ mol^−1^), l = depth of stomatal pore (m, 12 × 10 ^−6^ m for *C. australis*, mean of 10 replicates).

### Chlorophyll *a* fluorescence

The leaf chlorophyll fluorescence measurement was conducted using a PAM-2500 portable chlorophyll fluorometer (Walz, Effeltrich, Germany). The second fully expanded leaf of *S. speciosa* and the third leaf for *C. australis* and *F. benjamina* were selected for this measurement. Leaves were dark adapted with a leaf clip for 20 min, then a 0.6 s saturating light pulse (3,450 μmol m^−2^ s^−1^) was given to obtain the F_m_ and F_0_. After that, the leaf was light adapted with 5 min continuous actinic light (100 μmol m^−2^ s^−1^, similar as the growing condition) with saturating pulses every 25 s, after that, the maximum light adapted fluorescence (F_m_′) and steady state fluorescence (F_s_) were recorded. The maximum photochemical efficiency of PSII (F_v_/F_m_) was calculated as F_v_/F_m_ = (F_m_−F_0_)/F_m_ (Genty et al., 1989). Then, the actinic light was turned off and a far-red pulse was applied to obtain the minimal fluorescence after the PSI excitation (F_0_′). PSII operating efficiency (Φ_PSII_) was calculated as Φ_PSII_ = (F_m_′ − F_s_)/F_m_′ and qP was calculated as qP = (F_m_′ − F_s_)/(F_m_′ − F_0_′); NPQ, which is proportional to the rate constant of the thermal energy dissipation, was estimated as NPQ = (F_m_−F_m_′)/F_m_′ (Baker, 2008). The electron transport rate (ETR) was calculated as ETR = Φ_PSII_ × PAR × 0.84 × 0.5, where the absorbed photon energy (PAR) is assumed to be equally distributed between PSI and PSII and 0.84 is the assumed light absorbance of the leaf.

### Pigments content

Leaf chlorophyll content was determined according to Lichtenthaler and Buschmann (2001). One Hundred Fifty milligrams fresh leaf was grinded using liquid nitrogen and extracted in 80% acetone overnight at −20°C. Absorbance at 470 nm (A_470_), 645 nm (A_645_), and 663 nm (A_663_) was measured with a spectrophotometer (Infinite 200, Tecan Group Ltd., Switzerland) and the pigment contents were calculated from the following equations: Chl *a* = 12.25 × A_663_−2.79 × A_645_; Chl *b* = 21.50 × A_645_−5.10 × A_663_, and Carotenoids = (1000 × A_470_−1.82 × Chl *a*−85.02 × Chl *b*)/198.

### Plant growth measurements

The second fully expanded leaf area counting from the apex was measured using a leaf area meter (Li-Cor 3000, Li-Cor, USA) this in four replicates. Four plants per treatment and cultivar were used for the biomass measurements. After aerial fresh weight (FW) determination plants were oven-dried at 85°C for 3 days until a constant mass was reached to determine dry weight (DW).

### Statistical analysis

Data are presented as means ± SE. Data were analyzed for light quality for each species by one-way analysis of variance (ANOVA), after verifying homoscedasticity by Levene's test. Tukey's HSD test was used to compare means at *p* < 0.05. Correlations between traits were tested using Pearson's correlation coefficients. A regression testing K_leaf_ as function of leaf thickness and stomatal conductance was performed. All statistical analyses were conducted using SPSS Statistics 22 (IBM Software, Chicago, USA).

## Results

### Biomass and leaf characteristics

**Figure 2:**
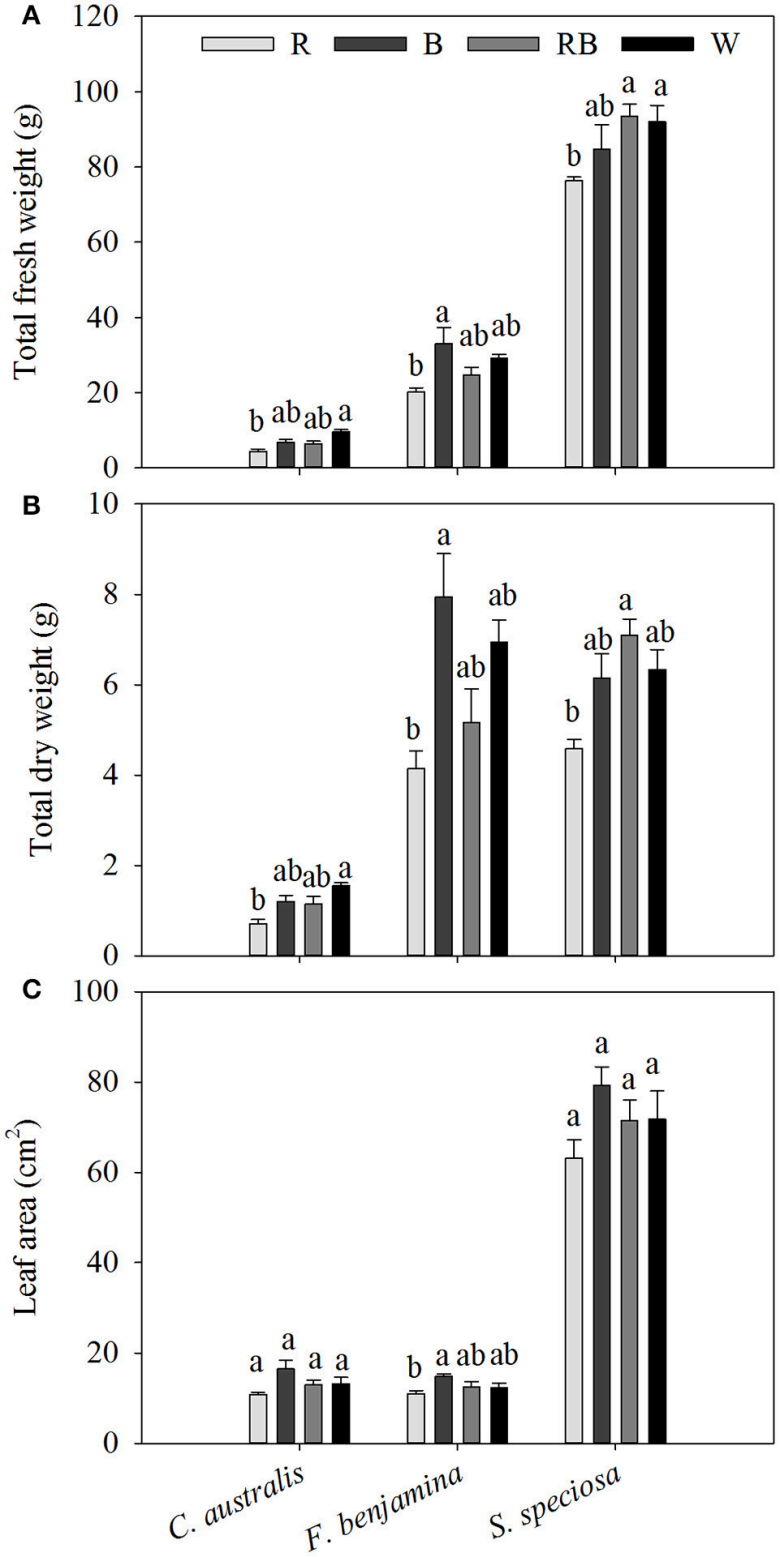
Effects of light quality on total aboveground fresh weight **(A)**, total dry weight **(B)** and individual leaf area **(C)** of *C. australis, F. benjamina*, and *S. speciosa*. Data are presented as means ± standard error (*n* = 4). Different letters indicate significant differences between values (*p* < 0.05).

In *C. australis*, total aboveground fresh weight was the greatest under W, followed by B and RB and significantly decreased under R, similar the dry weight was greatest under W and declined under R (Figure 2). Biomass (both FW and DW) of *F. benjamina* and *S. speciosa* were significantly lower under R, while no significant difference between the other light qualities were found.

The three species had very different leaf morphologies (Figure 2, Table 2). *C. australis* and *F. benjamina* had relative small leaves, while *S. speciosa* developed large leaves. B enhanced the leaf area of *F. benjamina* followed by RB and W while it significantly decreased under R. B tended to increase the individual leaf area in both *C. australis* and *S. speciosa* though this was not significant (*P* = 0.070 and 0.183, respectively).

**Table 2:** Effect of light quality on the leaf anatomy of leaves of *C. australis, F. benjamina*, and *S. speciosa*.

**Species**	**Light quality**	**Adaxial epidermis (μm)**	**Abaxial epidermis (μm)**	**Palisade parenchyma (μm)**	**Spongy parenchyma (μm)**	**Leaf thickness (μm)**
*C. australis*	R	10.06 ± 0.69b	12.25 ± 0.55a	/	/	168.97 ± 3.46c
	B	12.90 ± 0.56a	14.81 ± 0.69a	/	/	196.29 ± 0.78b
	RB	13.50 ± 0.39a	12.22 ± 0.54a	/	/	205.53 ± 1.42b
	W	12.63 ± 0.84ab	14.88 ± 0.75a	/	/	244.44 ± 3.29a
*F. benjamina*	R	28.10 ± 0.59c	18.58 ± 0.76ab	20.10 ± 1.41c	83.40 ± 3.99b	150.19 ± 3.88c
	B	45.95 ± 1.08a	20.97 ± 0.83a	35.68 ± 0.59a	127.63 ± 2.75a	230.28 ± 2.82a
	RB	43.64 ± 0.72ab	19.97 ± 0.90a	23.01 ± 0.63c	81.81 ± 3.43b	168.43 ± 4.62b
	W	40.33 ± 1.14b	16.69 ± 0.46b	27.31 ± 0.90b	95.14 ± 2.98b	179.46 ± 3.43b
*S. speciosa*	R	31.33 ± 0.92b	21.61 ± 1.81b	45.43 ± 2.16b	282.18 ± 17.64a	380.54 ± 18.65a
	B	46.85 ± 1.14a	32.94 ± 2.35a	53.70 ± 1.05a	264.56 ± 13.91a	398.05 ± 17.83a
	RB	42.12 ± 1.85a	25.03 ± 0.94b	57.13 ± 1.11a	280.44 ± 4.17a	404.71 ± 6.69a
	W	43.11 ± 0.99a	24.33 ± 1.55b	45.12 ± 1.49b	301.09 ± 9.54a	413.64 ± 8.95a

*Different letters indicate significant differences between values (p < 0.05) for each parameter. Data given as means ± SE (n = 5)*.

**Figure 3:**
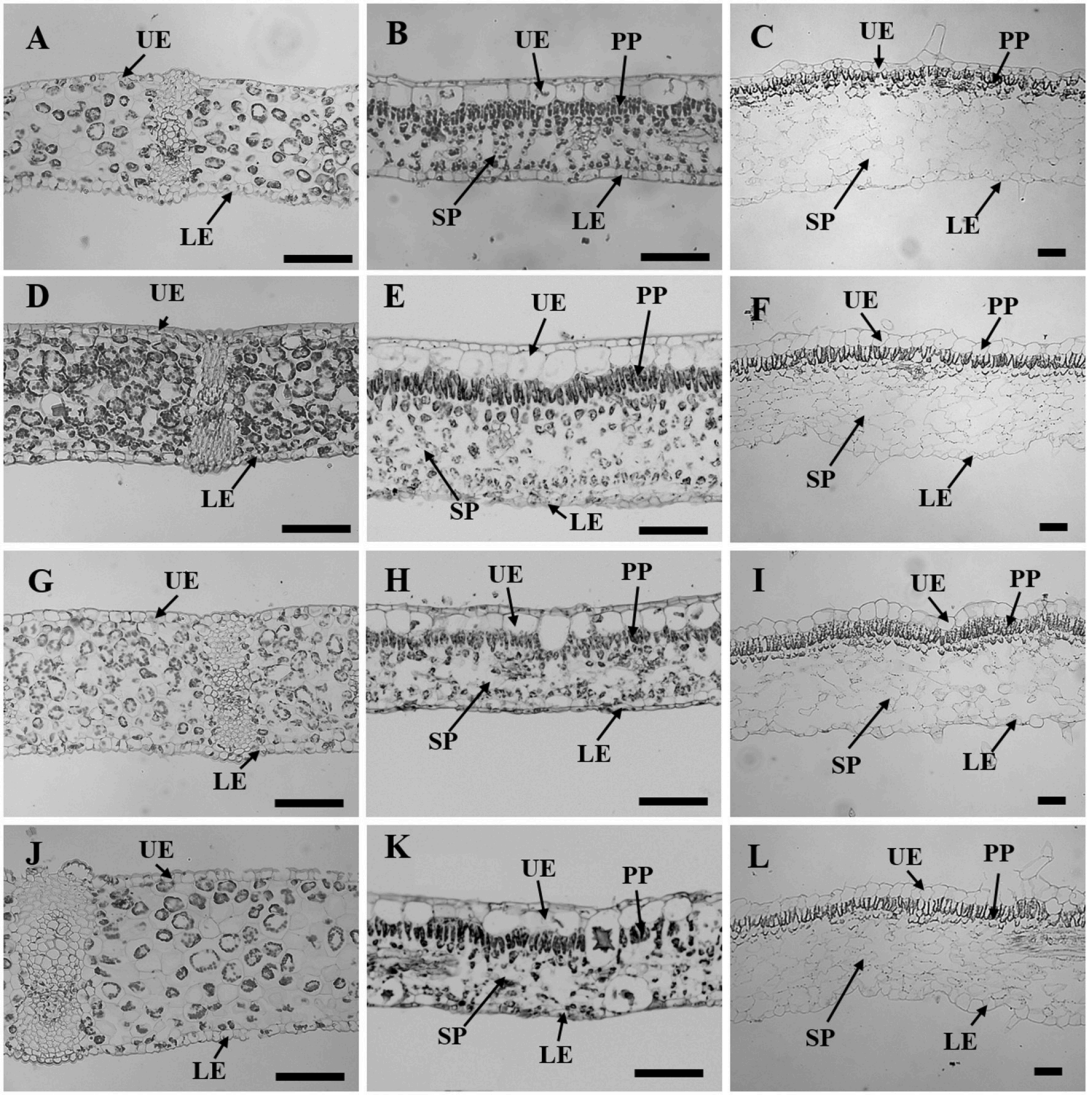
Leaf sectioning anatomy of *C. australis* (left panel), *F. benjamina* (middle panel), and *S. speciosa* (right panel) developed under Red light **(A–C)**, Blue light **(D–F)**, Red with Blue **(G–I)** and White **(J–L)**. Black bar = 100 μm. UE, upper epidermis; LE, lower epidermis; PP, palisade parenchyma; SP, spongy parechyma.

Leaf thickness in *C. australis* was highest under W followed by RB and B while the thinnest leaves were found under R (Table 2, Figure 3). As *C. australis* is a monocot, the leaf anatomy is isobilateral and the mesophyll is hardly differentiated into palisade and spongy parenchyma cells. Therefore, only the adaxial and abaxial epidermal thickness was measured which contribute, respectively, 6.1 ± 0.23 and 6.7 ± 0.26% of the total leaf thickness. Abaxial epidermis was not affected by light quality while the thinnest adaxial epidermis was found under R while B and RB had the thickest epidermal cells.

Leaf thickness in *F. benjamina* was greatest under B, lower under RB and W while it was significantly thinner under R (Table 2). *F. benjamina* has evergreen glossy leaves and the adaxial and abaxial epidermis contribute, respectively, 21.8 ± 1.0 and 10.6 ± 0.5% to the leaf thickness. Especially the adaxial epidermis is strongly reduced under R followed by W. The effect on the abaxial epidermis is not as strong though also here the thinnest cell layers are under R and W. The leaf thickness difference is strongly influenced by the mesophyll. In absolute value the palisade parenchyma is highest under B although it represents only 15.5% of the total leaf thickness while the palisade layer is, respectively, 26% under RB and 24% under W. B also strongly enhances the spongy parenchyma while it is not affected by the other light qualities. In *S. speciosa*, leaf thickness was not affected by the different light qualities (Table 2). *S. speciosa* has velvety hairy leaves and the adaxial and abaxial epidermis contribute, respectively, 10.2 ± 0.5 and 6.5 ± 0.4% to the leaf thickness. Adaxial epidermal thickness was found thinnest under R while it tended to be thicker under B though not significant differing from RB and W. Abaxial epidermis was thickest under B. Palisade parenchyma thickness was found lower under R and W and significantly greater under B and RB while no effect were found for the spongy parenchyma.

Leaf thickness correlated with Φ_PSII_ in *C. australis* (*r* = 0.855) but this correlation was weaker in *F. benjamina* (*r* = 0.622) while thickness of the palisade parenchyma correlated moderately with Φ_PSII_ in *S. speciosa* (*r* = 0.674).

### Leaf hydraulic conductance

**Figure 4:**
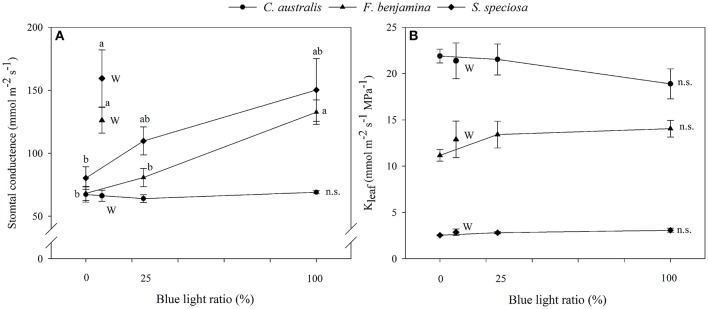
Effects of blue light ratio on stomatal conductance **(A)** and leaf hydraulic conductance **(B)** of *C. australis, F. benjamina*, and *S. speciosa*. Data are presented as means ± standard error (*n* = 4). Different letters indicate significant differences between values (*p* < 0.05) and n.s. indicates no significant differences. W indicates the multispectral white treatment.

Light quality tended to influence the leaf hydraulic conductance of the selected ornamentals though effects were not significant (Figure 4). In *C. australis* K_leaf_ was lowest under B and slightly increased under R, RB, and W. In *F. benjamina* and *S. speciosa* K_leaf_ was lowest under R and highest under B. On average K_leaf_ was highest in *C. australis*, followed by *F. benjamina* and quite low in *S. speciosa*.

Correlation study between K_leaf_ and other leaf characteristics showed positive correlations with leaf thickness and stomatal conductance in *F. benjamina* and *S. speciosa* (**Figure 6**). However, for the monocot *C. australis*, a negative trend with stomatal conductance was found and no correlation with leaf thickness.

### Stomatal characteristics and stomatal conductance

The effects of light quality on the stomatal characteristics are given in Table 3. The aperture length was not affected by light quality, this for the three species. An increase of aperture area was found in *C. australis* under B, while no effects were found in *F. benjamina* and *S. speciosa*. The width/length ratio was not affected by light quality (data not shown). Total aperture area per unit leaf area was not affected by light quality though it tended to be lower under R for *F. benjamina* and *S. speciosa*. Stomatal index and density were significantly affected by the light quality treatments. In *C. australis* stomatal index decreased under R though density was not affected. *C. australis* also showed the highest stomatal density of the studied ornamentals, as it ranged between 274.75 N° mm^−2^ under B up to 325.10 N° mm^−2^ under R. Likewise a high density of epidermal cells per unit leaf area was present but B significantly reduced the number of epidermal cells (Table 3). In *F. benjamina*, both R and B gave the lowest stomatal index while the highest index was found under W; the stomatal density was lowest under R and highest under W. In *S. speciosa* both the highest stomatal density and index were found under B and W and the lowest under R.

**Table 3:** Effect of light quality on the stomatal characteristics of leaves of *C. australis, F. benjamina*, and *S. speciosa*.

**Species**	**Light quality**	**Aperture length (μm)**	**Aperture width (μm)**	**Aperture area (μm^2^)**	**Total aperture area/ leaf area (cm^2^ m^−2^)**	**Stomatal index (%)**	**Stomatal density (N mm^−2^)**	**Epidermal cell density (N mm^−2^)**
*C. australis*	R	10.42 ± 0.71a	3.20 ± 0.22ab	26.34 ± 0.82b	88.32 ± 8.48a	18.39 ± 1.27b	325.10 ± 5.21a	1502.8 ± 33.9a
	B	11.42 ± 0.32a	3.76 ± 0.13a	33.72 ± 0.82a	92.52 ± 1.84a	23.62 ± 1.27a	274.60 ± 4.20a	1174.4 ± 44.0b
	RB	10.84 ± 0.18a	3.09 ± 0.12b	26.35 ± 1.17b	83.99 ± 3.99a	24.60 ± 1.48a	320.32 ± 17.91a	1315.5 ± 59.5ab
	W	11.09 ± 0.42a	3.28 ± 0.05ab	28.76 ± 1.51ab	87.50 ± 6.02a	24.08 ± 1.04a	304.36 ± 9.59a	1264.6 ± 38.4b
*F. benjamina*	R	12.64 ± 0.34a	5.18 ± 0.20ab	51.19 ± 2.07a	67.03 ± 7.45a	13.91 ± 0.67b	130.06 ± 10.18b	935.4 ± 33.4a
	B	13.05 ± 0.77a	5.73 ± 0.24a	58.82 ± 4.85a	83.99 ± 7.11a	15.42 ± 0.80b	143.55 ± 8.06ab	935.8 ± 17.8a
	RB	13.16 ± 0.27a	5.04 ± 0.19ab	52.02 ± 2.08a	86.22 ± 4.03a	19.04 ± 0.99ab	165.84 ± 4.99ab	877.9 ± 25.0ab
	W	12.72 ± 0.40a	4.81 ± 0.12b	48.18 ± 2.26a	83.66 ± 3.57a	22.03 ± 2.011a	175.50 ± 6.49a	799.1 ± 15.6b
*S. speciosa*	R	15.78 ± 0.83a	5.48 ± 0.16a	68.42 ± 3.83a	25.79 ± 3.03a	17.42 ± 0.72b	37.71 ± 1.88b	872.4 ± 27.4a
	B	18.57 ± 0.51a	6.23 ± 0.16a	91.51 ± 3.83a	46.93 ± 2.97a	25.84 ± 1.29a	51.22 ± 1.88a	805.6 ± 11.2a
	RB	18.22 ± 0.70a	6.89 ± 0.55a	99.83 ± 11.17a	43.04 ± 6.53a	21.10 ± 1.59ab	42.41 ± 2.56ab	815.2 ± 32.8a
	W	16.07 ± 1.16a	5.93 ± 0.30a	76.10 ± 9.17a	40.63 ± 7.05a	24.61 ± 1.64a	52.86 ± 4.14a	866.3 ± 40.2a

*Different letters indicate significant differences between values (p < 0.05) for each parameter. Data given as means ± SE (n = 5)*.

The stomatal conductance of the ornamentals was differentially affected by the different light qualities (Figure 4). For *C. australis*, no effects were noted on the stomatal conductance with respect to increasing B. For both *F. benjamina* and *S. speciosa* stomatal conductance increased with increasing B when comparing R, RB, and B. However, multispectral W yielded the highest stomatal conductance in both species. A strong correlation of stomatal density (*r* = 0.979) with g_s_ and stomatal index (*r* = 0.995) with g_s_ was found *in S. speciosa*.

### Chlorophyll *a* fluorescence

Effects of light quality on chlorophyll fluorescence parameters of the studied ornamentals are given in Table 4. The maximum quantum efficiency F_v_/F_m_, was influenced by the applied light quality and overall we saw a lower value of F_v_/F_m_ for R (*P* = 0.003). For *C. australis*, the lowest value was observed under R, F_v_/F_m_ increased under W and RB while B gave the highest F_v_/F_m_-value. For *F. benjamina* and *S. speciosa* F_v_/F_m_ declined under R compared to the other spectral qualities.

**Table 4:** Effect of light quality on chlorophyll fluorescence parameters: F_v_/F_m_, Φ_PSII_, qP, NPQ, and ETR of *C. australis, F. benjamina*, and *S. speciosa*.

**Species**	**Light quality**	**F_v_/F_m_**	**Φ_PSII_**	**qP**	**NPQ**	**ETR**
*C. australis*	R	0.536 ± 0.040c	0.349 ± 0.034b	0.791 ± 0.030b	0.303 ± 0.027c	13.75 ± 1.25b
	B	0.738 ± 0.009a	0.427 ± 0.019ab	0.814 ± 0.023ab	0.934 ± 0.091a	16.60 ± 0.75ab
	RB	0.702 ± 0.008ab	0.477 ± 0.014a	0.873 ± 0.007a	0.692 ± 0.048b	18.40 ± 0.60a
	W	0.654 ± 0.014b	0.479 ± 0.012a	0.887 ± 0.003a	0.440 ± 0.051c	18.60 ± 0.60a
*F. benjamina*	R	0.745 ± 0.005b	0.603 ± 0.024bc	0.898 ± 0.023a	0.270 ± 0.066a	15.80 ± 0.58bc
	B	0.792 ± 0.003a	0.677 ± 0.003a	0.941 ± 0.007a	0.272 ± 0.025a	17.80 ± 0.20a
	RB	0.785 ± 0.007a	0.662 ± 0.010ab	0.937 ± 0.011a	0.252 ± 0.011a	17.17 ± 0.31ab
	W	0.772 ± 0.006a	0.598 ± 0.011c	0.890 ± 0.006a	0.447 ± 0.109a	15.25 ± 0.48c
*S. speciosa*	R	0.628 ± 0.021b	0.358 ± 0.031b	0.786 ± 0.025b	0.666 ± 0.049a	13.60 ± 1.21b
	B	0.733 ± 0.011a	0.490 ± 0.041a	0.862 ± 0.023ab	0.582 ± 0.088ab	18.80 ± 1.66a
	RB	0.745 ± 0.010a	0.598 ± 0.007a	0.940 ± 0.007a	0.364 ± 0.028b	23.00 ± 0.32a
	W	0.749 ± 0.005a	0.520 ± 0.021a	0.877 ± 0.022a	0.605 ± 0.054a	19.80 ± 0.86a

*Different letters indicate significant differences between values (p < 0.05) for each parameter. Data given as means ± SE (n = 5)*.

Φ_PSII_, qP and ETR showed a similar reaction to the light quality treatments. For both *C. australis* and *S. speciosa* the lowest values for Φ_PSII_ were observed under R. For *F. benjamina*, Φ_PSII_ was significant higher under B, while R and W gave lower values. For both *C. australis* and *S. speciosa* highest qP were found for RB and W while no effect of light quality was found for *F*. *benjamina*.

NPQ significantly increased under B followed by RB compared to W and R in *C. australis*, while for *S. speciosa*, it significantly increased under R and W followed by B compared with RB. However, for *F. benjamina*, no effect of light quality was found on NPQ (*P* = 0.117), though it tended to be higher under W.

### Leaf pigment contents

**Figure 5:**
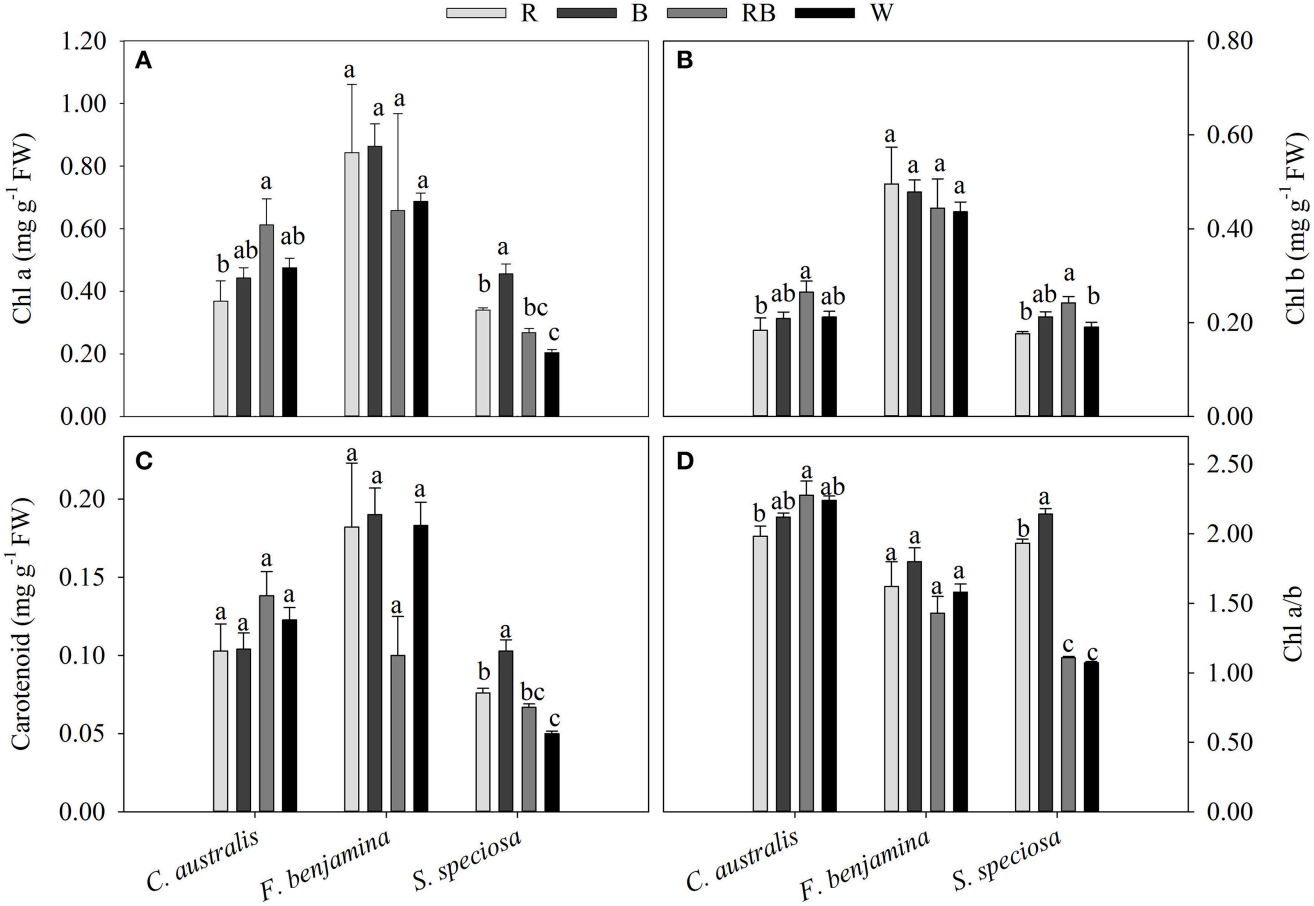
Effects of light quality on chlorophyll a and b **(A,B)** and carotenoid **(C)** content and Chl a/b ratio **(D)** of *C. australis, F. benjamina*, and *S. speciosa*. Data are presented as means ± standard error (*n* = 4). Different letters indicate significant differences between values (*p* < 0.05).

The total pigment content was different between the species (Figure 5). In *F. benjamina*, the total chlorophyll content ranged from 1.102 to 1.338 mg g^−1^, while it was 0.395 to 0.668 mg g^−1^ and 0.395 to 0.668 mg g^−1^ for *S. speciosa* and *C. australis*, respectively. The carotenoids were higher in *C. australis* (0.103–0.138 mg g^−1^) and *F. benjamina* (0.100–0.190 mg g^−1^) followed by *S. speciosa* (0.050–0.103 mg g^−1^). Overall the total chlorophyll content was not significantly affected by the light quality (*P* = 0.468) though there were species differences (Figure 5). In *C. australis* the highest Chl *a*, Chl *b*, and Chl *a*/*b* was found under RB and the lowest content was found under R, while no significant effect on carotenoid content was present. In *F. benjamina*, no significant effects of light quality on chlorophyll and carotenoid content were observed. Blue light yielded the highest Chl *a*, Chl *a*/*b*, and carotenoid content in *S. speciosa* leaves followed by R. The lowest Chl *a*, Chl *a*/*b*, and carotenoid content were found for W, this treatment lead to a decrease of 55 and 51% for Chl *a* and carotenoids compared to B.

## Discussion

Leaf photosynthesis requires the interception of light. Light inside the leaf is influenced by the wavelength, the light level and the angle of the incident light (Brodersen and Vogelmann, 2010) as well as by the leaf anatomy. Light is absorbed by chloroplasts while passing through the palisade and spongy mesophyll. The vertically elongated palisade cells minimize light scattering, allowing a deeper penetration, while spongy tissue enhances the light capture by scattering light (Evans, 1999). *F. benjamina* and *S. speciosa* are both dicots with palisade and spongy mesophyll. *F. benjamina* reacted strongly to B not only in total leaf thickness but also by an increasing effect on all anatomical structures. Reduction or absence of blue light decreased leaf thickness and respective anatomical structures and this was most pronounced for monochromatic R. This reaction reflects the observations on pepper (Schuerger et al., 1997) and wheat (Goins et al., 1997) where increased levels of B to R increased the palisade and spongy mesophyll thickness. In *S. speciosa*, however, total leaf thickness was not affected but a reorganization of the mesophyll resulting in a higher percentage of palisade parenchyma (16%) was observed for B and RB while for W and R the palisade parenchyma averaged 13% of the total mesophyll. The greater cell surface area per unit of mesophyll volume makes palisade tissue a more efficient structure in term of photosynthesis than spongy mesophyll (Evans, 1999). For the monocot *C. australis*, the full spectrum W resulted in the thickest leaves though comparing R with RB and B also indicated the favorable effect of B on leaf thickness.

**Figure 6:**
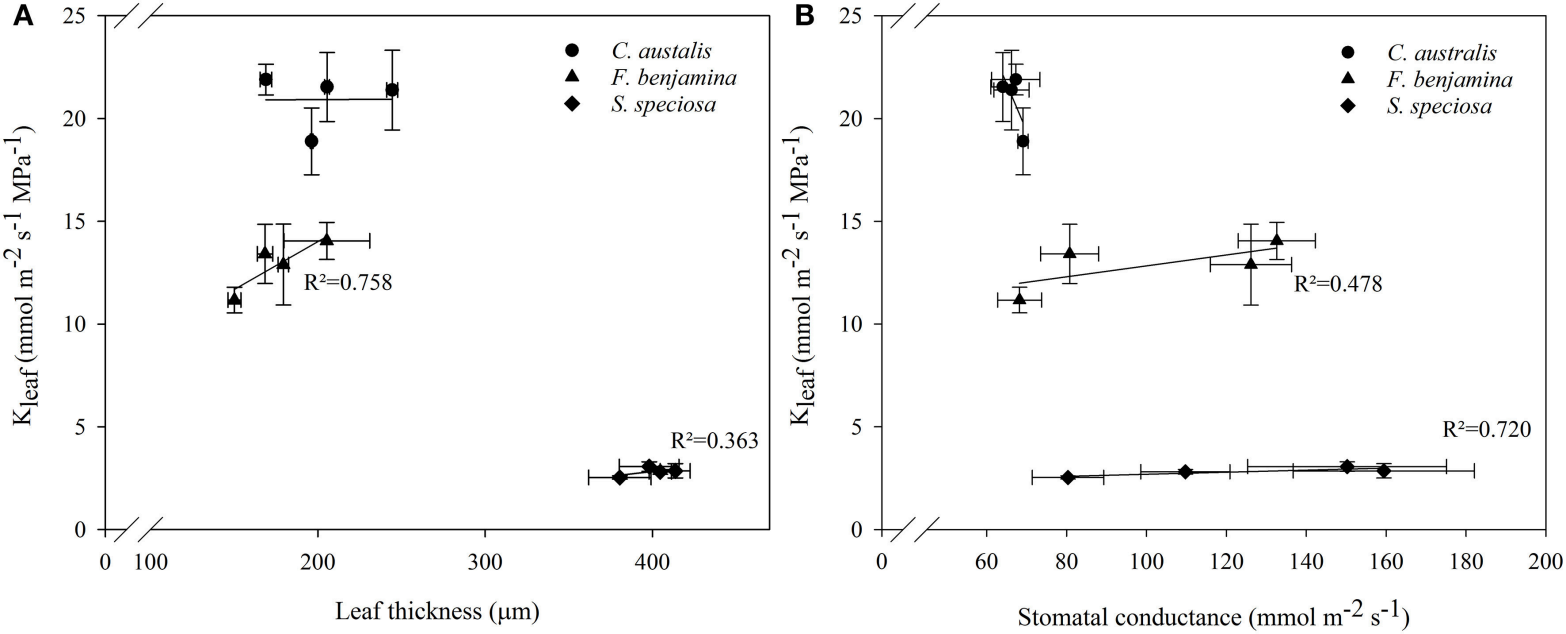
Correlations between leaf thickness and K_leaf_ **(A)** and stomatal conductance and K_leaf_ **(B)** of *C. australis, F. benjamina*, and *S. speciosa* under different light qualities. Values presents the mean of four replicates with standard errors (*n* = 4).

Schuerger et al. (1997) also reported an effect of blue light on secondary xylem formation in peppers suggesting an effect of light quality on water translocation. Buckley et al. (2015) suggested that greater leaf thickness should contribute to a higher leaf conductance (K_leaf_) given the greater number of parallel pathways for horizontal transport to the sites of evaporation, if those sites are distributed throughout the leaf. More specifically the maximal K_leaf_ correlated with palisade thickness, and palisade/spongy mesophyll ratio for tropical rainforest tree species (Sack and Frole, 2006). K_leaf_ of bur oak enhanced under blue and green light compared to other wavelengths (Voicu et al., 2008). However, in bur oak one focused mainly on short term responses to light quality while this study was conducted on leaves that were formed under a given spectral light quality. Therefore, effects on K_leaf_ can be attributed to differences in the development of leaf mesophyll and veins. K_leaf_ varied strongly between the studied species and was much greater in *C. australis* than in *S. speciosa*, while *F. benjamina* was intermediate (Figure 4). This variation in K_leaf_ among species is reported by several authors and can fluctuate up to 65-fold across plant species (Sack and Holbrook, 2006; Brodribb et al., 2012; Buckley, 2015). Under B, K_leaf_ of the dicots *F. benjamina* and *S. speciosa* tended to be higher. This is in agreement with Savvides et al. (2012), who were the first to report that cucumber leaves that developed under B and RB had a higher K_leaf_. Furthermore, K_leaf_ correlated with thickness of leaf (*r* = 0.79) and palisade parenchyma (*r* = 0.78) in *F. benjamina* as well as in *S. speciosa* (*r* = 0.46 and *r* = 0.50, respectively) (Figure 6). In contrast, we found quite different results in the monocot *C australis*, where K_leaf_ was independent of leaf thickness. The leaf anatomical structure of monocots makes that water in the major vein exits into surrounding tissue of bundle sheath cells instead of the minor veins (Xiong et al., 2015). We did not quantify leaf venation in this study although it might influence the leaf hydraulic conductance (Nardini et al., 2003). However, it is more likely that the small variations in both K_leaf_ and leaf thickness explain the absence of a relation in *C. australis*. K_leaf_ and g_s_ correlated positively (*r* = 0.48 and 0.72, respectively) in both *F. benjamina* and *S. speciosa* which agrees with previous observations (Augé et al., 2008; Brodribb et al., 2012; Savvides et al., 2012).

Stomatal development is influenced by light quality which in turn will influence the conductance (g_s_) of air through the leaf mesophyll and stomata. Blue light increased the stomatal density of chrysanthemum (Kim et al., 2004) and this was also observed in *F. benjamina* and *S. speciosa*. Moreover, additional blue light increased the stomatal index in all the studied species and both parameters (stomatal index and stomatal density) were highly correlated in *F. benjamina* and *S. speciosa* (*r* = 0.99 and 0.97, respectively). These results reflect the effect of blue light on the development of stomata, which is mediated through the additive function of CRY1 and CRY2 (Pillitteri and Torii, 2012). Stomatal density and index are not correlated in *C. australis* which is due to the lower stomatal density under blue (Table 3). In *C. australis* the total number of epidermal cells per unit of area was also reduced under B in comparison with monochromatic R, indicating larger epidermal cells under B. Likewise in *Pelargonium* leaves the positive effect of blue light on the elongation of epidermal cells was shown (Fukuda et al., 2008).

However, not only the stomatal density but the additive effect of the stomatal aperture influences the stomatal conductivity. It is well known that blue light affects stomatal opening through the photoreceptors phototropin and cryptochrome (Liscum et al., 2003; Shimazaki et al., 2007; Boccalandro et al., 2012). As a result of this blue light signaling, increased stomatal conductance if blue is added to red might be expected. Indeed, we found a positive effect if B was added to the R spectrum on the stomatal conductance in *F. benjamina* and *S. speciosa* (Figure 4). Likewise, blue light or addition of B to the spectrum enhanced the total aperture area per unit of leaf area in both *F. benjamina* and *S. speciosa* (Table 3) even though the correlations with g_s_ were not significant (*r* = 0.61 and 0.79, respectively). In cucumber, the decline of stomatal conductance under monochromic green, yellow and red light correlated also with reduced photosynthesis (Wang et al., 2009). However, we did not find significant correlations between g_s_ and Φ_PSII_ in *F. benjamina* and *S. speciosa*. The lower light intensities in this study (100 μmol m^−2^ s^−1^ compared to 350 μmol m^−2^ s^−1^ in cucumber) may indicate that we were still below the threshold of g_s_ to limit photosynthesis.

Chlorophyll content directly influences the photosynthetic potential as well as the primary production (Curran et al., 1990; Gitelson et al., 2003). Also the chlorophyll content is affected by the light quality and several studies showed the beneficial effect of blue in the light spectrum (Sæbø et al., 1995; Hoffmann et al., 2015). Long-term exposure of leaves to blue light enhances the 5-aminolevulinic acid synthesizing activity (Kamiya et al., 1983) which in turn mediates the biosynthesis of all tetrapyrroles such as hemes and chlorophylls. Also in our study B or RB was favorable for chlorophyll content in *S. speciosa* and *C. australis* though this effect was not very strong. For *F. benjamina* no effects on chlorophyll content were found. This differential response might be due to species effects as also Lin and Hsu (2004) found no effect on pigment content in lettuce leaves.

Different wavelengths penetrate differently into the leaf, blue and red are efficiently absorbed close to the surface, whereas green light contributes more to photosynthesis in deeper leaf layers (Sun et al., 1998; Brodersen and Vogelmann, 2010). In spinach leaves blue light was almost completely absorbed at 300 μm leaf depth while red tailored to 400 μm and green light to 600 μm leaf depth (Evans, 1999). This reflects the more effective absorption of blue light by chlorophyll (Terashima et al., 2009). Thicker leaves and thicker palisade parenchyma will result in a better absorbance and therefore higher photosynthetic yield (Hanba et al., 2002; Haliapas et al., 2008; Shengxin et al., 2016). The decrease in leaf mesophyll thickness by red light led to a lower photosynthetic yield and photochemical quenching (Tables 2, 4), so leaf thickness did contribute to the higher photosynthetic performance under B and RB in this study. The reduced Φ_PSII_ in *F. benjamina* under W (leaf thickness = 179.46 μm) compared to B (leaf thickness = 230.28 μm) might be explained by the partial absorbance of the green wavelengths which were not captured by the photosynthetic pigments (Fankhauser and Chory, 1997) though we did not observe this in the monocot species, *C. australis*.

Irrespective of the penetration depths of light the applied light quality strongly influenced the photosynthetic efficiency (F_v_/F_m_′ Φ_PSII_) and R had a significant negative effect in the three species. This negative effect of monochromatic R was already reported in cucumber (Wang et al., 2009; Savvides et al., 2012), despite the fact that R coincides with the absorbance peak of chlorophyll and is known for its higher relative quantum efficiency than B in the instantaneous photosynthetic response (McCree, 1971). Tennessen et al. (1994), however, showed that long term monochromatic R causes an imbalance of photons available to Photosystem I and Photosystem II. Long term absence of blue light reduces the photosynthetic performance which is known as the “red light syndrome” (Trouwborst et al., 2016). This leads to photo-damage as shown by the reduced F_v_/F_m_ in this experiment. The effects of additional blue light on photosynthetic performance are integrated in the produced plant biomass which was lowest under R in the three species while no significantly differences in B, RB, and W were found.

## Conclusion

We show here for the first time how narrow-band R, B, and RB modulates leaf morphology, mesophyll anatomy, stomatal formation and hydraulic conductance of leaves of *C. australis, F. benjamina*, and *S. speciosa* in comparison with broad spectrum white-LEDs.

Blue light enhanced leaf thickness in *C. australis* and *F. benjamina* and palisade parenchyma thickness in *S. speciosa* which suggest a better light absorption for this treatment. Adding blue to red light increased the stomatal index in the three species and enhanced the total aperture per leaf unit in *F. benjamina* and *S. speciosa*. Although, K_leaf_ was not significantly affected by light quality a moderate correlation between K_leaf_ and leaf thickness and K_leaf_ and stomatal conductance was found for both dicot species *F. benjamina* and *S. speciosa* though not for the monocot *C. australis*.

Leaves of the three species that developed solely under red light were characterized by a lower F_v_/F_m_ and Φ_PSII_ indicating a malfunctioning of photosynthesis which also resulted in a lower dry mass production under red. The chlorophyll fluorescence parameters of the other three light treatments (B, RB, and W) were hardly influenced and also the dry weight production was not influenced.

## Author contributions

LZ and MV conceived and designed the experiments. LZ performed the experiments, analyzed the data and drafted the manuscript, MV critically revised the manuscript. All authors reviewed and approved the final manuscript.

### Conflict of interest statement

The authors declare that the research was conducted in the absence of any commercial or financial relationships that could be construed as a potential conflict of interest.
